# Inhibition of STING alleviates lipotoxicity in viral-infected primary mouse hepatocytes and viral hepatitis-associated liver damage

**DOI:** 10.3389/fphar.2026.1804426

**Published:** 2026-04-28

**Authors:** Yize Zhang, Yifei Li, Yingqiao Qin, Shaomei Liang, Kunpeng Liu, Xue Liang

**Affiliations:** 1 Gene Hospital of Henan Province, The First Affiliated Hospital of Zhengzhou University, Zhengzhou, China; 2 Guangxi Key Laboratory of Special Biomedicine, School of Medicine, Guangxi University, Nanning, China; 3 Guangxi Academy of Medical Sciences, The People’s Hospital of Guangxi Zhuang Autonomous Region, Nanning, China

**Keywords:** lipid droplets, liver damage, liver injury, STING, viral infection

## Abstract

**Introduction:**

RNA virus infections often cause liver steatosis and lipid deposition, which significantly exacerbate liver damage. However, the underlying mechanisms linking viral-induced lipid accumulation to hepatocellular death remain incompletely understood.

**Methods:**

We employed a primary mouse hepatocyte model of vesicular stomatitis virus (VSV) infection and an in vivo mouse model of virus-induced acute liver failure. The role of STING was investigated through genetic knockdown and pharmacological inhibition using H-151. Furthermore, clinical relevance was assessed using data from the National Health and Nutrition Examination Survey (NHANES, 2007–2010) to analyze correlations between hepatic steatosis and injury biomarkers.

**Results:**

Viral exposure induced time-dependent upregulation and activation of STING, which driven the pathological accumulation of lipid droplets and subsequent ferroptotic cell death. Genetic knockdown or pharmacological inhibition of STING (H-151) effectively mitigated metabolic stress and preserved cell viability. These protective effects were recapitulated in vivo, where H-151 administration significantly improved survival and reduced tissue damage in a mouse model of virus-induced acute liver failure. Analysis of NHANES data corroborated these findings, showing that in patients with concurrent viral hepatitis and metabolic dysfunction-associated steatotic liver disease (MASLD), the fatty liver index (FLI) and triglyceride levels correlate linearly with alanine aminotransferase (ALT) and aspartate aminotransferase (AST) levels.

**Conclusion:**

Collectively, our study identifies the STING-lipotoxicity axis as a key driver of viral hepatotoxicity, highlighting its potential as a therapeutic target for liver injury.

## 1 Introduction

RNA virus infections frequently cause significant disruptions in host lipid metabolism. Clinical data show that hepatic steatosis is highly prevalent in patients with chronic hepatitis C virus (HCV), occurring in 40%–80% of cases (Rubbia-Brandt et al., 2000; Asselah et al., 2006). Similarly, HIV-monoinfected individuals exhibit a higher prevalence of MASLD than the general population, at approximately 35% (Maurice et al., 2017). HCV induces lipid accumulation through several complex mechanisms. The viral core protein inhibits the activity of microsomal triglyceride transfer protein (MTP), which impairs the secretion of very-low-density lipoproteins (VLDL) (Perlemuter et al., 2002; Mirandola et al., 2006). This core protein also localizes to the surface of lipid droplets to disrupt lipid homeostasis (Barba et al., 1997) and increases *de novo* lipogenesis by upregulating fatty acid synthase (FAS) (Jackel-Cram et al., 2007). Additionally, HCV infection impairs fatty acid oxidation pathways mediated by PPARα (Dharancy et al., 2005) and induces hypobetalipoproteinemia (Serfaty et al., 2001). Although the role of steatosis as an independent driver of liver fibrosis remains a subject of debate (Asselah et al., 2006; Leandro et al., 2006), it is widely accepted that steatosis significantly increases the severity of liver injury (Adinolfi et al., 2001). This exacerbation is primarily characterized by the promotion of hepatocyte death by accumulated lipids (Kumar et al., 2002; Walsh et al., 2004). However, the specific molecular mechanisms through which viral-induced steatosis triggers cell death have not yet been fully determined.

Accumulating evidence indicates that the cGAS-STING pathway is essential for the innate immune response against RNA virus infections (Hu and Shu, 2023; Jahun et al., 2023). The primary mechanism by which RNA viruses activate STING involves the induction of mitochondrial stress, leading to the translocation of mitochondrial DNA (mtDNA) into the cytosol. For instance, the M2 protein of influenza A virus (IAV) and the 2B protein of encephalomyocarditis virus (EMCV) both function as viroporins that facilitate mtDNA leakage, thereby triggering STING-dependent antiviral immunity (Moriyama et al., 2019; Liu et al., 2023). Additionally, the IAV PB1-F2 protein has been shown to drive mtDNA release by inducing mitochondrial cristae collapse and reverse electron transport, which activates the cGAS-STING-NF-κB signaling axis (Cheung et al., 2024). Together, these findings suggest that RNA virus infections activate STING through the release of mtDNA, an endogenous damage-associated molecular pattern (DAMP), to induce antiviral gene expression. Considering that our previous research demonstrated that STING activation enhances mTOR activity to inhibit lipophagy and lead to the abnormal accumulation of intracellular LDs (Liu et al., 2022), we aimed to investigate whether this STING-mTOR-lipid axis also drives the progression of viral-induced liver damage. However, our previous research demonstrated that STING activation enhances mTOR activity, which subsequently inhibits lipophagy and leads to the abnormal accumulation of intracellular lipid droplets (Liu et al., 2022). Based on these observations, the present study aims to investigate whether STING activation similarly leads to pathological lipid accumulation in hepatocytes during RNA virus infection. Furthermore, we explore how this virus-induced metabolic remodeling influences antiviral efficacy and the ultimate progression of liver damage.

The cGAS-STING pathway serves as a central axis for sensing ectopic cytosolic DNA and initiating innate immune responses. In the canonical activation model, cGAS recognizes cytosolic DNA to catalyze the synthesis of the second messenger cyclic GMP-AMP (cGAMP) from GTP and ATP. cGAMP subsequently induces the oligomerization of STING at the endoplasmic reticulum (ER) and its translocation to the ER-Golgi intermediate compartment (ERGIC) and the Golgi apparatus (Hu and Shu, 2023; Burdette and Vance, 2013). During this translocation, STING recruits and activates the downstream kinase TBK1, which phosphorylates the transcription factor IRF3. This process ultimately drives the transcription of type I interferons and various antiviral effector genes (Burdette and Vance, 2013; Hu and Shu, 2023). Beyond cytokine production, the non-canonical roles of STING in regulating lipid homeostasis are increasingly being recognized. Our previous study demonstrated that activated STING further enhances mTORC1 activity via the TBK1 signaling axis under metabolic stress (Liu et al., 2022). As a critical negative regulator of autophagy, overactivated mTOR significantly suppresses lipophagy, thereby impairing the lysosomal degradation of triglycerides. This STING-mediated inhibition of autophagy leads to the pathological accumulation of lipid droplets in hepatocytes, which triggers lipotoxic damage due to the increased availability of substrates for lipid peroxidation.

In this study, we observed that infection with VSV induces progressive mortality in primary mouse hepatocytes. This cytotoxicity was characterized by a time-dependent upregulation of STING protein expression and activation., Corroborating our previous findings that STING signaling drives mTOR activation (Liu et al., 2022), we noted a concurrent rise in mTOR activity following infection. Crucially, this heightened STING-mTOR signaling coincided with intracellular lipid droplet accumulation and increased cell death. STING inhibitor H-151 attenuated both lipid deposition and cytotoxicity in primary mouse hepatocytes, suggesting a causal link. Validating these findings *In Vivo*, H-151 treatment effectively ameliorated hepatic injury in a mouse model of VSV-induced acute liver failure. The clinical relevance of this metabolic disruption is further supported by data from the National Health and Nutrition Examination Survey (NHANES). In patients with comorbid viral hepatitis and MASLD, hepatic steatosis (as assessed by the fatty liver index, FLI) and triglyceride levels correlated linearly with injury markers (ALT and AST), implying that dysregulated lipid metabolism exacerbates viral liver damage. Collectively, these results elucidate the interplay between STING activation, metabolic dysregulation, and hepatocyte viability, highlighting STING inhibition as a promising therapeutic strategy for liver injury.

## 2 Materials and methods

### 2.1 Cells and virus

Primary hepatocytes were freshly isolated from live mice. The procedure involved a two-step collagenase perfusion technique. Mice were first perfused with Ca^2+^and Mg^2+^-free Hanks’ balanced salt solution (HBSS; Gibco, 14185052) containing EGTA (Sigma, E4378) at 2.5 mM via portal vein cannulation (Step 1). This was followed by perfusion with an enzymatic solution containing collagenase P (Sigma, 11249002001) (Step 2). The resulting cell suspensions were filtered through a 70 μm nylon mesh, centrifuged at 50 *g* for 1 min at 4 °C, and the collected cell pellets, consisting of hepatocytes, were used for subsequent experiments. Primary hepatocytes were cultured in Dulbecco’s modified Eagle medium (DMEM; Gibco, C11995500BT). The medium was enriched with 10% fetal bovine serum (Gibco, 1600044), 1% GlutaMAX Supplement (Gibco, 35050061) and 1% penicillin-streptomycin (Invitrogen, 15140122). Cultures were maintained at 37 °C in a humidified atmosphere containing 5% CO_2_. VSV (Indiana strain) was sourced from our laboratory stocks. VSV is a prototypic enveloped, negative-strand RNA virus of the Rhabdoviridae family. It is widely utilized as a standard model in virology and immunology due to its rapid replication cycle, broad host range, and its ability to robustly induce innate immune responses, particularly the type I interferon system.

### 2.2 Virus infection *In Vivo*

The study involved 18 six-week-old C57BL/6 mice, which were randomly allocated into control and experimental groups (6 mice per group). Mice were maintained at a controlled room temperature (25 °C) with a standardized light/dark cycle. For *In Vivo* virus infection, the VSV virus was propagated in Vero cells and subsequently concentrated via sucrose gradient centrifugation. Mice were subjected to direct intraparenchymal liver injection of VSV (1 × 10^6^ pfu in a total volume of 50 µL per mouse), and then administered either vehicle or H-151 (10 mg/kg/day) via intraperitoneal injection for 5 consecutive days. Survival of infected mice was monitored daily until day 7 post-infection and analyzed using Graphpad Prism 8.3. At the termination of the experiment, mouse livers were collected for histopathological assessment of tissue damage. All experimental procedures were conducted in accordance with the Animal Care and Use Regulations of China, and were approved by the Animal Ethics Committee of the People’s Hospital of Guangxi Zhuang Autonomous Region (Approval No. KY-GZR-2025-008). The study adhered to the ARRIVE guidelines for reporting *In Vivo* experiments.

### 2.3 NHANES study

**FIGURE 1:**
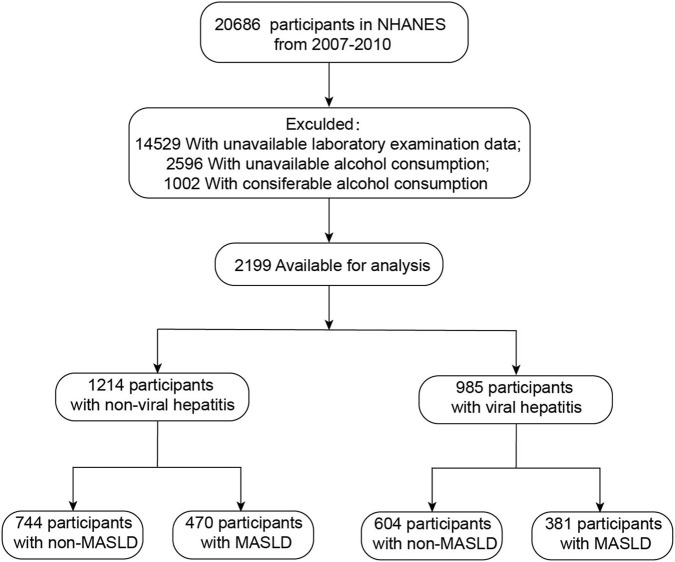
Flowchart of the sample selection from NHANES 2007–2010.

The National Health and Nutrition Examination Survey (NHANES) aims to assess the health and nutritional status of the United States population. NHANES uses a complex, multi-stage probability design and operates in 2-year cycles to collect information on the civilian, non-institutionalized U.S. population. This cross-sectional study used the NHANES data from 2007 to 2010 and included adults aged 20 years and above. A total of 20686 participants were screened. Participants were first divided into viral hepatitis and non-viral hepatitis groups based on the presence or absence of viral hepatitis. Furthermore, each group was then further divided into MASLD and non-MASLD subgroups. MASLD was defined by the fatty liver index (FLI). Exclusion criteria were: (1) missing laboratory data (n = 14529); (2) missing alcohol consumption data (n = 2596); (3) excessive alcohol intake (>3 drinks/day for men or >2 drinks/day for women) (n = 1002). After exclusions, 2199 participants remained for analysis (Figure 1).

Demographic, physical, and laboratory data were collected. (1) Demographics: age, sex, race/ethnicity, education, and alcohol use. Race/ethnicity was coded as Mexican American, non-Hispanic White, non-Hispanic Black, other Hispanic, or other. Education was classified as below high school, high school or equivalent, or college and above. Alcohol use was recorded as none or light-to-moderate. (2) Physical measures: body-mass index (BMI) and waist circumference. BMI was calculated as weight in kilograms divided by height in meters squared. (3) Laboratory measures: aspartate aminotransferase (AST), alanine aminotransferase (ALT), γ-glutamyl transferase (GGT), and triglycerides (TG).

The fatty liver index (FLI) was computed with the equation below: FLI = (e^0.953∗ln (TG)+0.139∗BMI+0.718∗ln (GGT)+0.053∗waist circumference−15.745^)/(1 + e^0.953∗ln (TG)+0.139∗BMI+0.718∗ln (GGT)+0.053∗waist circumference−15.745^) ∗ 100(PMID: 17081293). An FLI ≥60 was taken to indicate MASLD.

The analysis followed the NHANES sampling scheme and used the provided survey weights to produce nationally representative estimates. Categorical variables are presented as unweighted counts with weighted proportions and continuous variables as medians with interquartile ranges. Spearman’s rank correlation was used to assess linear associations. All calculations were performed in DecisionLinnc software (Team, 2023), and two-sided *P* < 0.05 was considered statistically significant.

### 2.4 Antibodies and reagents

The primary antibodies utilized in the experiment included anti-STING1 (Proteintech, 19851-1-AP; Proteintech, 66680-1-Ig; CST, 13647) monoclonal anti-ACTB/ACTIN beta (Abclonal, AC026), anti-p-TBK1 (S172; Cell Signaling Technology [CST], 5483S), anti-TBK1 (CST, 38066S), and anti-GFP (ET1702-69, Huabio), along with p-p70S6K (CST, 9205S) and p70S6K (CST, 2708S). For Western blotting, the secondary antibodies employed were goat anti-rabbit IgG-HRP (CST, 7074S) and goat anti-mouse IgG-HRP (CST, 7076S). The chemical reagents were as follows: H-151 (aladdin, H287635) and BODIPY-493/503 (Invitrogen, D3922).

### 2.5 Transfection of siRNA

For siRNA transient transfection, hepatocytes were plated. LipoRNAiMAX (Invitrogen, 13778150) was used for transfection according to the manufacturer’s instructions. The sequences of siRNAs are listed as follows:

Scramble-siRNA: 5′- UUC​UCC​GAA​CGU​GUC​ACG​UTT −3′
*mSting*-siRNA-1#: 5′- GCA​UCA​AGA​AUC​GGG​UUU​ATT −3′
*mSting*-siRNA-2#: 5′- CAA​CAU​UCG​AUU​CCG​AGA​UTT −3′

### 2.6 qPCR

Total RNA was extracted using TRIzol reagent (Invitrogen, 15596026). Reverse transcription of total RNA to cDNA was performed with the Prescript RT reagent kit (Vazyme, R233-01). Real-time quantitative PCR was carried out using the LightCycler 480 Real-Time PCR System (Roche) and SYBR Green PCR Master Mix (Genstar, A301). Normalization was performed using the mGapdh reference genes. All primers used in this study are listed as fallow:

mSting-Forward: GGT​CAC​CGC​TCC​AAA​TAT​GTA​G
mSting-Reverse: CAG​TAG​TCC​AAG​TTC​GTG​CGA
mGapdh-Forward: TGT​GTC​CGT​CGT​GGA​TCT​GA
mGapdh-Reverse: GCT​TCA​CCA​CCT​TCT​TGA​T
mGpx4-Forward: GCC​TGG​ATA​AGT​ACA​GGG​GTT
mGpx4-Reverse: CAT​GCA​GAT​CGA​CTA​GCT​GAG
mAcsl4-Forward: CCT​GAG​GGG​CTT​GAA​ATT​CAC
mAcsl4-Reverse: GTT​GGT​CTA​CTT​GGA​GGA​ACG
mTrf-Forward: GCT​GTC​CCT​GAC​AAA​ACG​GT
mTrf-Reverse: GTC​ACG​GAA​GCT​GAT​GCA​CT
mTfrc-Forward: CCG​ACA​ATA​ACA​TGA​AGG​CTA​GT
mTfrc-Reverse: TTC​AGC​CAG​TTT​CAC​ACA​CTC

### 2.7 Quantification of mt-DNA release

Quantification of mt-DNA release was referred to previous studies (Liu et al., 2022). In briefly, a mitochondrial isolation kit (Beyotime, C3601) was used for the extraction of the cytoplasm of cultured primary hepatocytes. Collected hepatocytes were homogenized with 10-30 strokes, and was then centrifuged at 600xg for 10 min at 4 °C. The residual was used to extract the nuclear DNA. The collected supernatants were centrifuged at 11,000 g at 4 °C for 10 min. The residual was used to extract the mitochondria DNA. The final supernatants obtained from the centrifugation were considered as the cytosolic fraction without mitochondria, and were used to extract the released mt-DNA. DNA was isolated from the collected cytoplasmic fractions and mitochondria using a Cell-free DNA Isolation Kit with Magnetic Beads (Beyotime, D0085S). For mt-DNA copy number analysis: mt-mNd1 were amplified for determined the mt-DNA release by using real-time PCR. Normalization was performed using the mRpph1 qualification of collected mitochondria. All primers used in this study are listed as fallow:

mMt-nd1-Forward: TAT​CTC​AAC​CCT​AGC​AGA​AA
mMt-nd1-Reverse: TAA​CGC​GAA​TGG​GCC​GGC​TG
mRpph1-Forward: GGA​GAG​TAG​TCT​GAA​TTG​GGT​TAT​GAG
mRpph1-Reverse: CAG​CAG​TGC​GAG​TTC​AAT​GG

### 2.8 Cytotoxicity assay

The viability of primary mouse hepatocytes was analyzed using the CCK-8 kit (Dojindo, CK04) according to the manufacturer’s instructions. Primary mouse hepatocytes were plated into 96-well plates at a density of 3 × 10^4^ cells per well and incubated overnight. Subsequently, 10 μL of CCK8 reagent was added to each well, and the plates were incubated for 30 min at 37 °C in a cell culture incubator. Absorbance was measured at 450 nm using a multifunctional enzyme marker (Molecular Devices, SpectraMax i3x).

The mortality of primary mouse hepatocytes was analyzed using the cytotoxicity LDH Assay Kit (MCE, HY-K1090-100T) according to the manufacturer’s instructions. Primary mouse hepatocytes were plated into 96-well plates at a density of 3 × 10^4^ cells per well and incubated overnight. Add 10 μL of Lysis Solution to the high-control and high-control blank wells, and add 10 μL of medium to the low-control wells. The plates were incubated for 30 min at 37 °C in a cell culture incubator. Add 50 μL of Working Solution to each well for 30 min at room temperature. After incubation, add 50 μL of Stop Solution to each well and measure the absorbance at 490 nm using a a multifunctional enzyme marker (Molecular Devices, SpectraMax i3x).

### 2.9 Western blot analysis

Protein samples were resolved by SDS-PAGE electrophoresis and electrotransferred onto polyvinylidene fluoride (PVDF) membranes (Millipore, IPVH00010). The membranes were blocked with 5% skim milk for 1 h at room temperature to prevent nonspecific binding. Subsequent incubation with specific primary antibodies was carried out overnight at 4 °C, followed by incubation with suitable secondary antibodies. The protein expression was detected by using ChemiDoc XRS system (Bio-Rad Laboratories, Hercules, CA, United States).

### 2.10 Immunofluorescence

Cells were fixed with 4% paraformaldehyde for 15 min, followed by permeabilization in 100% methanol for 10 min. Blocking buffer was used for 1 h at room temperature. Primary antibodies were incubated with the cells overnight at 4 °C. After washing with PBS, the cells were incubated with fluorescently labeled secondary antibodies for 1 h. Nuclei were stained with Hoechst 33342 (Sigma, B2261). Fluorescence imaging was performed using a confocal laser scanning microscope (Zeiss, LSM880+Airyscan, Germany) or Inversed Fluorescent Microscope (Nikon, Ti2-U, Japan).

### 2.11 Triglyceride analysis

The levels of intracellular and hepatic triglyceride (TG) were quantified using a triglyceride assay kit (Applygen, E1030). The levels of liver enzymes ALT (SOLARbio, BC1555) and AST (SOLARbio, BC1565) in mouse serum were measured following the manufacturer’s protocol.

### 2.12 Hematoxylin and eosin (H&E) staining

H&E staining was performed to evaluate the severity of liver damage. Specifically, liver tissues were fixed in 4% paraformaldehyde (PFA), followed by dehydration and paraffin embedding according to standard histological protocols. Tissue sections were processed and stained with hematoxylin and eosin (H&E) to visualize histopathological alterations in liver tissue. Observations were conducted using an epi-fluorescence microscope (Nikon ECLIPSE Ti2, Japan).

### 2.13 Assessment of glutathione (GSH)/oxidized glutathione (GSSG) ratio

Primary mouse hepatocytes were seeded into 96-well plates. After infection with VSV and treatment with or without H-151 (10 µM) at 37 °C for 48 h, the cells were collected to measure the GSH/GSSG ratio. A commercial GSH/GSSG Ratio Detection Assay Kit (Abcam, ab138881) was utilized to determine the intracellular redox status, following the protocols provided by the manufacturer.

### 2.14 Malondialdehyde (MDA) measurement

Primary mouse hepatocytes were harvested and lysed. The cell lysates were mixed with thiobarbituric acid (TBA) and incubated at 100 °C for 15 min to form an MDA-TBA adduct. After the samples cooled to room temperature, the absorbance at 532 nm was measured using an Infinite M Nano plate reader. The final MDA levels were normalized to the total protein concentration and expressed as nmol/mg protein. The concentration of MDA was measured using a commercial Lipid Peroxidation MDA Assay Kit (Beyotime, S0131M) to evaluate lipid peroxidation.

### 2.15 Statistical analysis

Data processing was performed using GraphPad Prism 8.0 software. Quantitative results in histograms are presented as Mean ± SD. Relevant data were analyzed using Student’s t-test or one-way ANOVA. *P* < 0.05 was considered statistically significant.

## 3 Results

### 3.1 STING expression is lower in hepatocytes than non-parenchymal cells in mice liver

**FIGURE 2:**
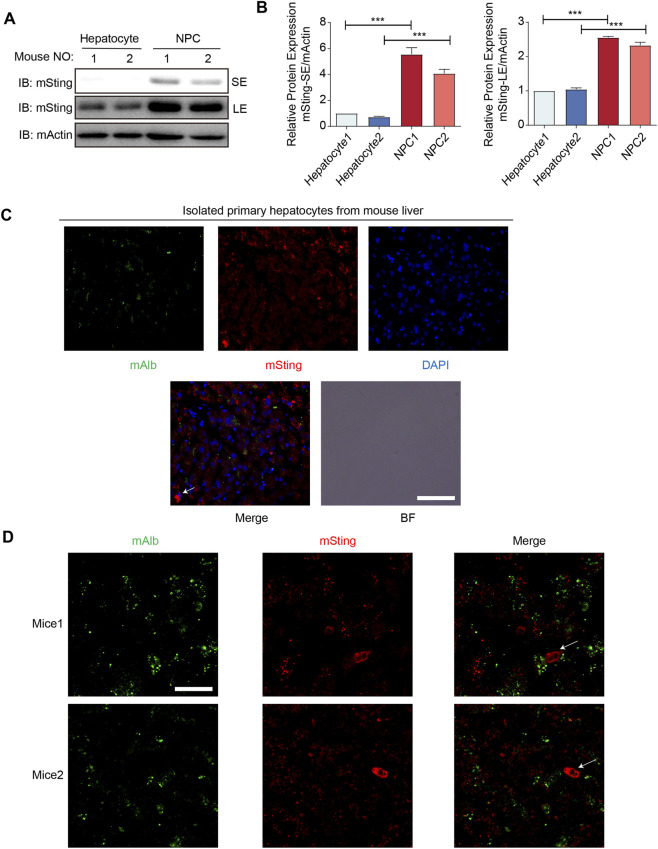
STING is weakly expressed in hepatocytes compared with NPCs. **(A,B)** Immunoblot analysis of STING in NPC or primary hepatocyte isolated from mice liver. SE: short exposure, LE: long exposure. **(C)** Immunofluorescence analysis of *mSting* fluorescence of liver from normal mice. Scale bar: 100 μm. **(D)** Immunofluorescence analysis of STING fluorescence of liver from normal mice. Scale bar: 100 μm ***p < 0.001, **p < 0.01, *p < 0.05. ns = not significant. Data are presented as mean ± SD from three independent experiments. Statistical significance between two groups was determined using Student’s t-test.

STING is a key signaling molecule in the innate immune system, primarily involved in cellular immune responses to DNA viruses and bacterial infections. Its activation is typically initiated by cGAS, a cytosolic DNA sensor, which subsequently activates TANK-binding kinase 1 (TBK1) via phosphorylation, ultimately leading to the expression of type I interferons (Burdette and Vance, 2013). Recent studies have demonstrated that in addition to mediating immune responses, STING is also involved in various forms of cell death, including apoptosis, necroptosis, and ferroptosis. However, the expression of STING in hepatocytes and its role in hepatocyte ferroptosis remain incompletely elucidated. To clarify the expression levels of STING in hepatocytes, we isolated primary hepatocytes and non-parenchymal cells (NPCs, primarily including Kupffer cells, hepatic stellate cells, and endothelial cells) from mouse liver. STING protein levels were analyzed by Western blot. The results showed that STING protein expression was significantly decreased in hepatocytes compared to NPCs (Figures 2A,B). This observation is highly consistent with the Human Protein Atlas (HPA) database (https://www.proteinatlas.org/ENSG00000184584-STING1/tissue), which reports that in the healthy human liver, STING1 protein is primarily localized to Kupffer cells and sinusoidal endothelial cells, while its expression in hepatocytes remains at low or undetectable levels. This reinforces the idea that STING’s basal activity is strictly regulated in hepatocytes under physiological conditions.

To further confirm the differential expression of STING, we performed immunofluorescence staining to observe its spatial distribution in hepatocytes and NPCs. Albumin (ALB) was used as a hepatocyte marker. In isolated total cells derived from mice liver, STING fluorescence signals were predominantly co-localized with NPCs, indicating low STING expression in hepatocytes but high expression in NPCs (Figure 2C). A similar pattern was observed in healthy mice liver tissues (Figure 2D). These findings indicate that STING is expressed at low levels in hepatocytes, suggesting that its basal expression and activity may be strictly regulated in these cells.

### 3.2 STING expression and activation increases in primary mouse hepatocytes following VSV infection

**FIGURE 3:**
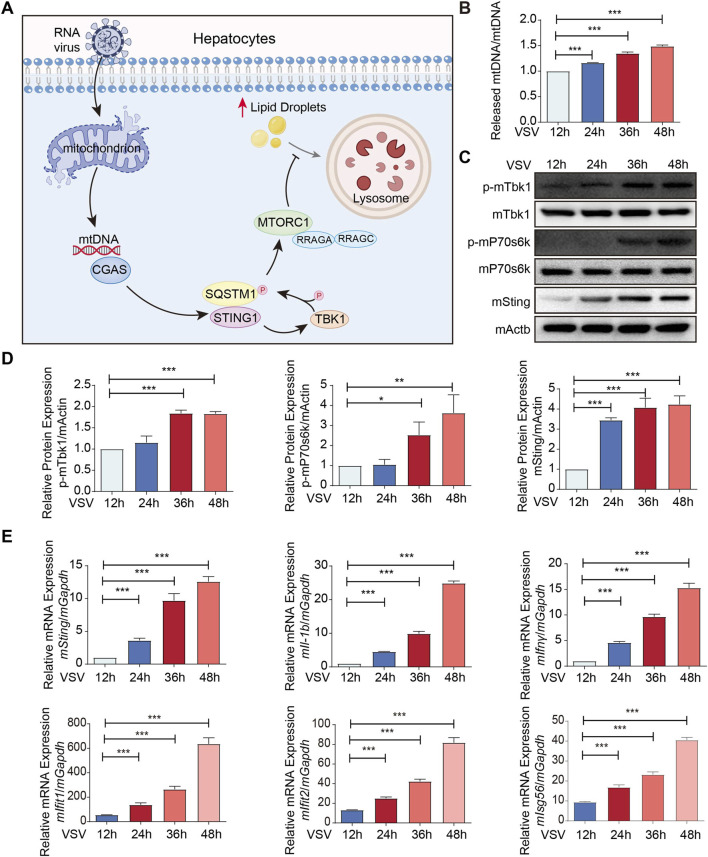
STING expression and activation increases in primary mouse hepatocytes following VSV infection. **(A)** The schematic depicts the mechanism by which RNA virus infection induces the release of mtDNA into the cytosol of hepatocytes. The leaked mtDNA subsequently activates the STING-TBK1-mTOR signaling axis, promoting intracellular LDs accumulation and the development of lipotoxicity. **(B)** qPCR analysis of released mt-DNA/total mt-DNA ratio in primary hepatocyte isolated from mice liver infected with VSV for 12 h/24 h/36 h/48 h. **(C,D)** Immunoblot analysis of p-mTbk1, p-mP70s6k and mSting in primary hepatocyte isolated from mice liver infected with VSV for 12 h/24 h/36 h/48 h. **(E)** qPCR analysis of *mSting*/*mIl-1b*/m*Ifnγ*/*mIfit1*/*mIfit2*/*mIsg56* mRNA in primary hepatocyte isolated from mice liver infected with VSV for 12 h/24 h/36 h/48 h. ***p < 0.001, **p < 0.01, *p < 0.05. ns = not significant. Data are presented as mean ± SD from three independent experiments. Statistical analysis was performed using one-way ANOVA.

As reported in previous studies, RNA viruses like VSV can trigger mitochondrial stress, leading to the leakage of mtDNA into the cytosol, which subsequently activates the cGAS-STING pathway to amplify the innate immune response (PMID: 25642965). So, we investigated the dynamic changes of STING and its downstream signaling molecule TBK1 in primary mouse hepatocytes at various time points post-VSV infection. In order to clarify the temporal activation of the STING-TBK1 axis during primary hepatocyte culture, we isolated hepatocytes from C57BL/6 mice and infected with VSV for 12 h/24 h/36 h/48 h. Our previous research demonstrated that the leakage of hepatic mitochondrial DNA (mtDNA) triggers the STING-TBK1-mTOR signaling pathway, leading to intracellular lipid droplet accumulation, enhanced lipotoxicity, and cell death (Figure 3A) (Liu et al., 2022). This study further confirmed that with extended VSV infection time, the isolated primary hepatocytes exhibited mtDNA release (Figure 3B).

Western blot assay was performed to assess the protein expression of mSting and phosphorylation levels of mTbk1 (p-mTbk1). The protein levels of mSting, p-mTbk1 and p-mP70s6k increased in a time-dependent manner from 12 to 48 h post-VSV infection. Conversely, total mTBK1 and mP70s6K protein levels remained largely unaltered throughout the course of VSV infection (Figures 3C,D). These data demonstrate that the STING-TBK1 signaling axis is robustly activated following VSV infection in primary mouse hepatocytes. Consistently, qPCR analysis revealed a time-dependent upregulation of *mSting*, *mIl-1b* and *mIfnγ* mRNA levels throughout the infection period. Furthermore, the expression of downstream interferon-stimulated genes (ISGs), including *mIfit1, mIfit2* and *mIsg56*, was also significantly increased, confirming the functional activation of the STING pathway (Figure 3E). Collectively, these data demonstrate that STING activation progressively intensifies in primary mouse hepatocytes following VSV infection, a process that may be associated with LDs accumulation and subsequent lipotoxicity-driven cell death.

### 3.3 The upregulation of STING is accompanied by an increase in the number of intracellular LDs in primary mouse hepatocytes following VSV infection

**FIGURE 4:**
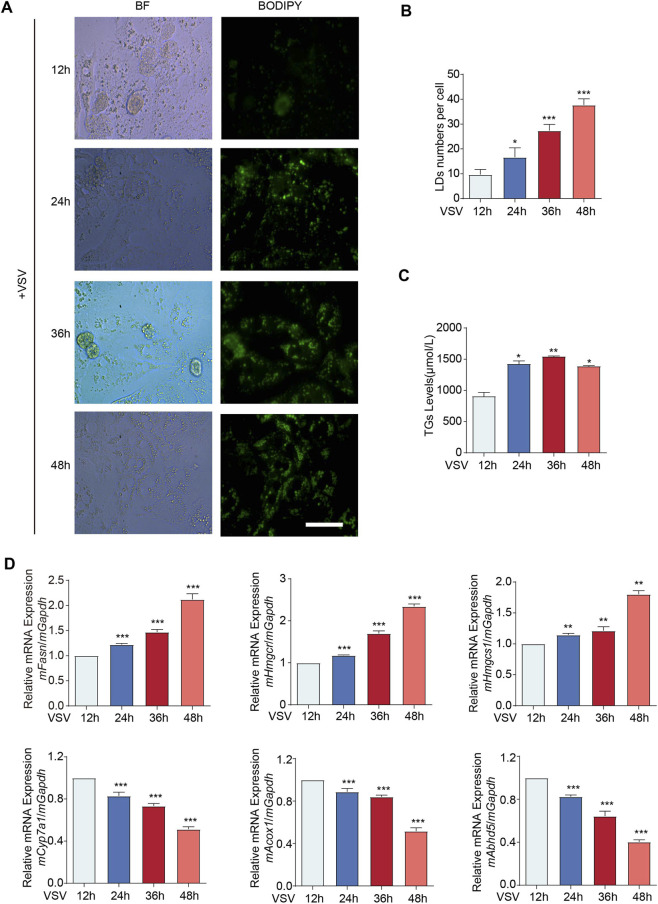
The upregulation of STING is accompanied by an increase in the number of intracellular LDs in primary mouse hepatocytes following VSV infection. **(A)** Primary mouse hepatocytes infected with VSV for 12 h/24 h/36 h/48 h were stained with BODIPY for LDs. Immunofluorescence analysis of the amount of GFP fluorescence in primary mouse hepatocytes. Scale bar: 20 μm. **(B)** Quantitative analysis of LD numbers per cell. Data were collected from at least five random fields for each group to support the visual observations in panel **(A,C)** Triglyceride assay kit analysis of the TG levels in primary mouse hepatocytes infected with VSV for 12 h/24 h/36 h/48 h. **(D)** qPCR analysis of *mFasn*/*mHmgcr*/*mHmgcs1*/*mCyp7a1*/*mAcox1*/*mAbhd5* mRNA in primary mouse hepatocytes infected with VSV for 12 h/24 h/36 h/48 h. ***p < 0.001, **p < 0.01, *p < 0.05. ns = not significant. Values represent mean ± SD from three independent experiments. Statistical significance was assessed by one-way ANOVA.

LDs serve as specialized intracellular organelles dedicated to the sequestration of neutral lipids, primarily triacylglycerols and cholesteryl esters. Imbalances in their biogenesis and degradation are implicated in the pathogenesis of metabolic disorders such as MASLD. Emerging evidence suggests that LD accumulation not only reflects metabolic dysfunction, but also participates in regulated cell death pathways, particularly ferroptosis, an iron-dependent form of non-apoptotic cell death characterized by lipid peroxidation and membrane integrity compromise (Pope and Dixon, 2023). Furthermore, STING,as a pivotal mediator of innate immune signaling, has recently emerged as a critical regulator of lipid metabolism and oxidative stress, potentially orchestrating the functional nexus between LD dynamics and ferroptotic signaling (Chen et al., 2025). To investigate the molecular link between STING induction and LD homeostasis, primary hepatocytes were isolated from C57BL/6 mice for functional assays. Intracellular LD accumulation was monitored and quantified via staining with the lipophilic fluorescent probe BODIPY. Immunofluorescence analysis demonstrated a marked temporal increase in LD abundance within these cultured hepatocytes; specifically, a progressive accumulation was observed between 12 and 48 h following VSV challenge, suggesting a correlation between viral infection and lipid remodeling (Figures 4A,B). Concomitantly, quantitative biochemical assays revealed a significant elevation in intracellular triglyceride (TG) content following VSV challenge, thereby corroborating the trends observed in our fluorescence imaging (Figure 4C). To further investigate the metabolic basis of this accumulation, we evaluated the expression profiles of key enzymes involved in lipid and cholesterol homeostasis. The results demonstrated that the mRNA levels of fatty acid synthase (*mFasn*) and critical cholesterol biosynthetic enzymes (*mHmgcr* and *mHmgcs1*) were progressively upregulated throughout the course of the infection. Conversely, the expression of *mCyp7a1*, a key enzyme in cholesterol catabolism, alongside fatty acid oxidation-related genes such as *mAcox1* and *mAbhd5*, was significantly downregulated following VSV infection (Figure 4D). These transcriptional profiles are highly consistent with the observed accumulation of LDs and TG in primary mouse hepatocytes. Such collective evidence points toward a concerted cellular response in primary mouse hepatocytes, where viral infection orchestrates a concurrent rise in STING expression and lipid storage, suggesting that STING may act as a pivotal regulator of metabolic rewiring during the innate immune response.

### 3.4 STING mediates the exacerbation of cell death and ferroptosis in primary mouse hepatocytes during prolonged VSV infection

**FIGURE 5:**
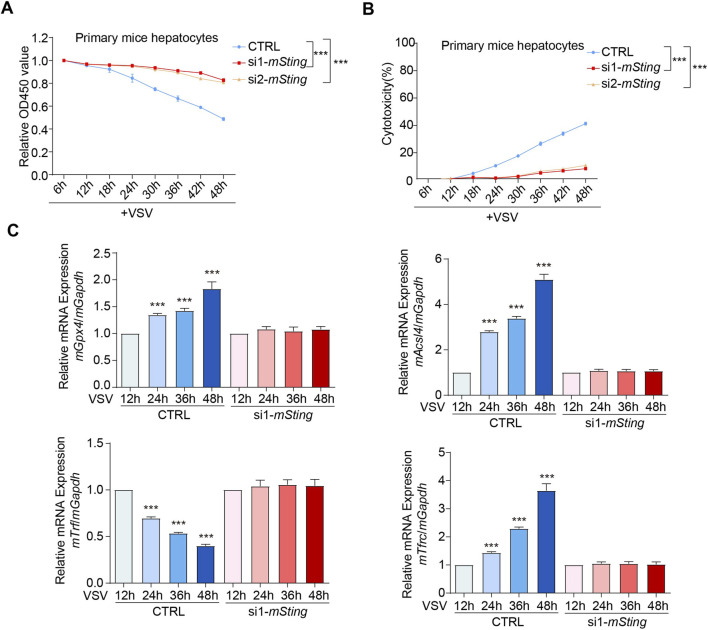
The enhancement of ferroptosis increases in primary mouse hepatocytes during prolonged VSV infection. **(A)** CCK8 assay analysis of the viability of the CTRL or si1/2-*mSting* primary mouse hepatocytes infected with VSV for 6h–48 h. **(B)** LDH assay analysis of the cytotoxicity of the CTRL or si1/2-*mSting* primary mouse hepatocytes infected with VSV for 6h–48 h. **(C)** qPCR analysis of *mGpx4/mAcsl4/mTrf/mTrfc* mRNA in the CTRL or si1-*mSting* primary mouse hepatocytes infected with VSV for 12 h/24 h/36 h/48 h. ***p < 0.001, **p < 0.01, *p < 0.05. ns = not significant. Values represent mean ± SD from three independent experiments. Statistical analysis was performed using one-way ANOVA.

Ferroptosis represents a distinct mode of iron-dependent regulated cell death, driven by the toxic accumulation of lipid peroxides and membrane compromise. Increasing evidence implicates this pathway in the etiology of diverse pathologies, ranging from hepatic injury and neurodegeneration to cancer. In this study, we investigated the temporal dynamics of ferroptosis in primary hepatocytes following VSV infection, longitudinally assessing cell viability, cytotoxicity, and the transcriptional profiles of key ferroptosis-associated genes. Assessment of cell viability via CCK-8 assay revealed a progressive, time-dependent decline following VSV infection. In the control group, viability dropped by approximately 50% at 36 h relative to the 6 h baseline. Conversely, hepatocytes transfected with si1/2-*mSting* exhibited significantly preserved viability, maintaining levels consistently higher than the control group across the 6–48 h interval (Figure 5A). Corroborating the viability data, cytotoxicity was further assessed via lactate dehydrogenase (LDH) release. In the control group, VSV infection triggered a progressive escalation in cell death, with cytotoxicity levels reaching approximately 40% by the 48 h time point. Conversely, this viral-induced damage was significantly mitigated in the si1/2-*mSting* group, which exhibited markedly lower LDH release throughout the prolonged infection period (Figure 5B). Mechanistically, qPCR analysis revealed that the expression of ferroptosis-associated markers (GPX4, ACSL4, and TFRC) was markedly induced during the course of VSV infection, accompanied by a suppression of transferrin (TRF) expression (Figure 5C). Notably, STING knockdown abrogated these infection-driven perturbations, restoring gene expression to near-baseline levels. Taken together with our viability and cytotoxicity findings, these results implicate STING-mediated ferroptosis as a primary driver of hepatocyte death during prolonged viral challenge, highlighting the protective role of STING inhibition.

### 3.5 The STING inhibitor H-151 reduces LDs accumulation in primary mouse hepatocytes during prolonged VSV infection

**FIGURE 6:**
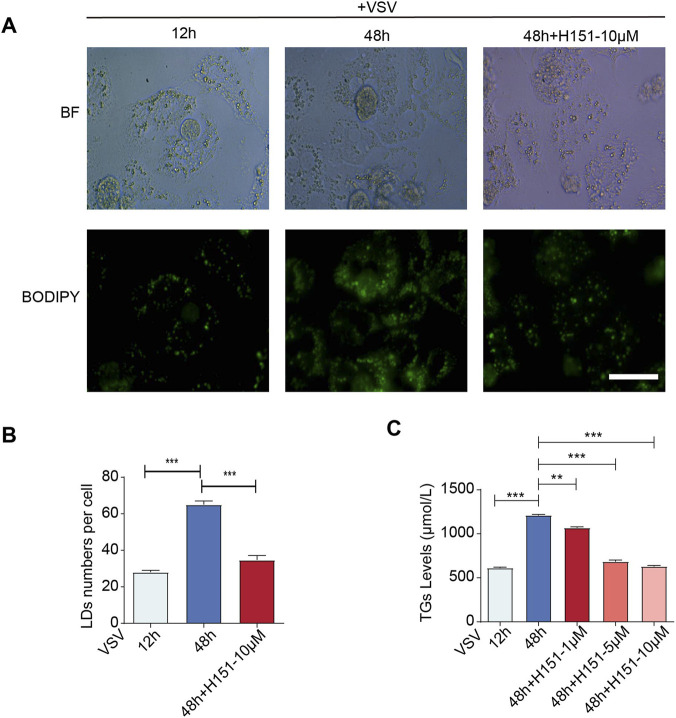
STING inhibition reduced the intracellular LDs in primary mouse hepatocyte during prolonged VSV infection. **(A)** Primary mouse hepatocytes infected with VSV were stained with BODIPY for LDs. Immunofluorescence analysis of the amount of GFP fluorescence in mouse primary hepatocytes treated with or without H-151 (10 μM). Scale bar: 20 μm. **(B)** Quantification of LD numbers per cell in primary hepatocytes treated with or without H-151. At least five independent fields were analyzed per group. **(C)** TG assay kit analysis of the TG levels in VSV-infected primary mouse hepatocytes treated with H-151 (1/5/10 μM). ***p < 0.001, **p < 0.01, *p < 0.05. ns = not significant. Data are presented as mean ± SD from three independent experiments. Statistical significance was determined using one-way ANOVA.

Given that the accumulation of LDs provides the substrate for lethal lipid peroxidation in ferroptosis, we investigated whether STING signaling governs this metabolic alteration in hepatocytes. By employing the specific STING inhibitor H-151 during VSV infection, we assessed LD dynamics via BODIPY staining. Fluorescence imaging revealed that pharmacological blockade of STING significantly attenuated the density of LDs compared to untreated controls (Figures 6A,B). This observation was corroborated by biochemical quantification, which demonstrated a dose-dependent reduction in intracellular TG levels upon H-151 treatment (Figure 6C). Collectively, these findings identify STING activation as a critical driver of aberrant lipid storage during viral infection.

### 3.6 The STING inhibitor H-151 attenuates ferroptosis-associated cellular damage in primary mouse hepatocytes during prolonged VSV infection

**FIGURE 7:**
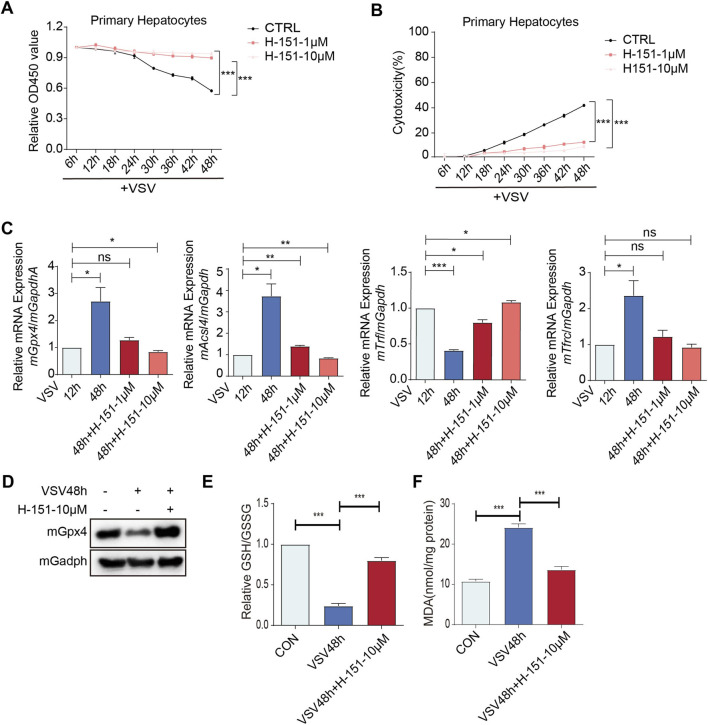
STING inhibition reduced the cell death in primary mouse hepatocytes during prolonged VSV infection. **(A)** CCK8 assay analysis of the viability of the primary mouse hepatocytes infected with VSV for 6 h–48 h, and treated with or without H-151 (1/10 μM). **(B)** LDH assay analysis of the cytotoxicity of primary mouse hepatocytes infected with VSV for 6 h–48 h, and treated with or without H-151 (1/10 μM). **(C)** qPCR analysis of *mGpx4/mAcsl4/mTrf/mTrfc* mRNA in primary mouse hepatocytes infected with VSV for 12 h/48 h, and treated with or without H-151 (1/10 μM). **(D)** Immunoblot analysis of mGpx4 protein expression in primary mouse hepatocytes infected with VSV for 48 h and treated with or without H-151 (10 µM). **(E)** Assessment of the relative GSH/GSSG ratio in primary mouse hepatocytes infected with VSV for 48 h and treated with or without H-151 (10 µM). **(F)** MDA were determined in primary mouse hepatocytes infected with VSV for 48 h and treated with or without H-151 (10 µM). ***p < 0.001, **p < 0.01, *p < 0.05. ns = not significant. Data are presented as mean ± SD from three independent experiments. Statistical significance was determined using one-way ANOVA.

We subsequently examined whether STING inhibition mitigates ferroptosis-associated cellular injury. In primary mouse hepatocytes challenged with VSV, administration of the STING inhibitor H-151 resulted in a marked preservation of cell viability (Figure 7A) and a concurrent reduction in cell death rates (Figure 7B). To substantiate the link to ferroptosis, we quantified the transcriptional levels of specific markers, including GPX4, ACSL4, and TFRC. As illustrated in Figure 7C, prolonged VSV infection (48 h) induced the upregulation of these genes. However, H-151 treatment significantly attenuated this response. These data collectively suggest that blocking STING activity dampens the activation of ferroptotic machinery, thereby protecting hepatocytes from infection-induced death. To further investigate the execution of ferroptosis at the protein and biochemical levels, we assessed Gpx4 expression and markers of oxidative stress. Western blot analysis showed that VSV infection significantly reduced Gpx4 protein levels, whereas H-151 treatment preserved Gpx4 expression (Figure 7D). We also measured the relative GSH/GSSG ratio to evaluate the cellular redox status. The results showed that VSV infection caused a severe depletion of the antioxidant pool, which was effectively reversed by STING inhibition (Figure 7E). Furthermore, the concentration of Malondialdehyde (MDA), a major indicator of lipid peroxidation, was significantly increased following VSV infection but was markedly reduced in the H-151 treatment group (Figure 7F). These findings indicate that the STING pathway promotes hepatocellular death by driving lipid peroxidation and impairing antioxidant defenses.

### 3.7 The STING inhibitor H-151 alleviates virus-induced liver injury in mice

**FIGURE 8:**
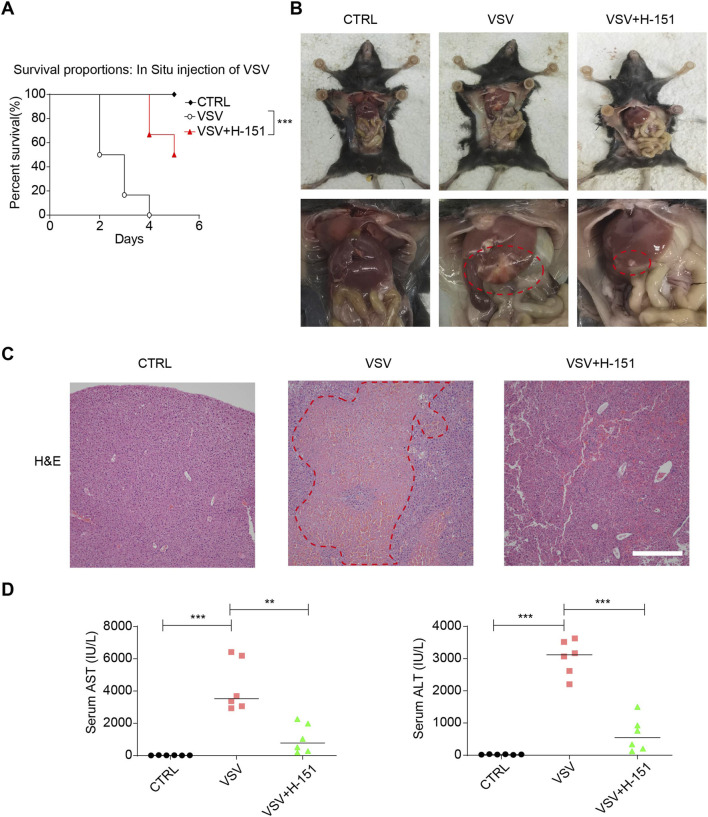
STING inhibition alleviated virus-induced liver damage in mice. **(A)** Mouse survival was monitored (n = 6). **(B)** Mouse dissection and liver imaging. **(C)** Hematoxylin and eosin staining of liver sections of mice. Scale bars = 200 μm. **(D)** Mouse serum was collected by orbital bleeding 7 days post-infection and used to measure the levels of liver enzymes ALT/AST. For survival analysis in **(A)**, the Log-rank (Mantel-Cox) test was used (n = 6 mice per group), ***p < 0.001 vs. VSV group. For **(D)**, data are mean ± SD (n = 6); statistical significance was determined using one-way ANOVA, ***p < 0.001, **p < 0.01, *p < 0.05. ns = not significant.

We established a mouse model of liver injury induced by intrahepatic injection of VSV to evaluate whether intraperitoneal administration of the STING inhibitor H-151 could mitigate VSV-induced hepatic damage. The results showed that H-151 treatment significantly prolonged the survival of mice with VSV-induced liver injury (Figure 8A). Upon necropsy, livers from VSV-infected mice exhibited extensive white lesions indicative of severe hepatic damage. In contrast, mice treated with H-151 showed a marked reduction in the area of white lesions, suggesting that it has partial protective effect on VSV-induced liver injury (Figure 8B). Similarly, hematoxylin and eosin (H&E) staining revealed large pale eosinophilic regions in the livers of VSV-infected mice, representing areas of tissue damage. H-151 treatment significantly reduced these pale eosinophilic regions, further confirming the alleviation of liver injury severity (Figure 8C). Serum levels of alanine aminotransferase (ALT) and aspartate aminotransferase (AST), commonly used markers of liver injury, were also assessed via retro-orbital blood collection. The results demonstrated that H-151 treatment significantly suppressed the VSV-induced elevation of serum ALT and AST levels (Figure 8D). In conclusion, these findings indicate that pharmacological inhibition of STING effectively alleviates VSV-induced liver injury in mice.

### 3.8 Hepatic lipid accumulation correlates with liver injury in patients with viral hepatitis and MASLD

**FIGURE 9:**
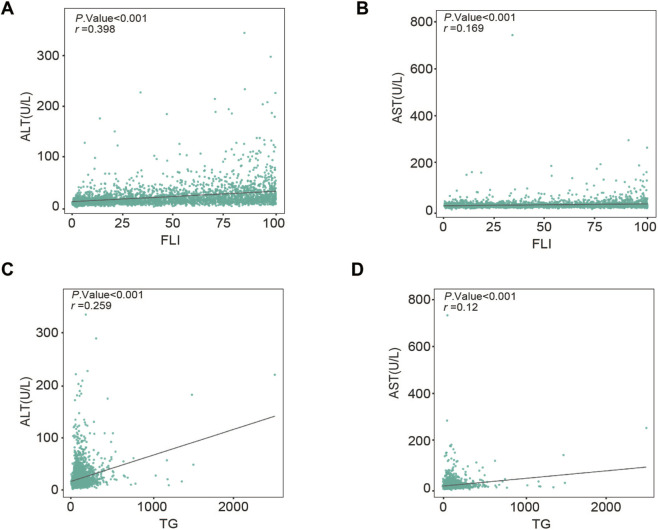
Linear positive associations between liver enzymes and hepatic lipid accumulation in patients with viral hepatitis and MASLD. **(A,B)** Correlation of ALT and AST with FLI. **(C,D)** Correlation of ALT and AST with TG. Statistical associations were analyzed using Spearman’s rank correlation. The correlation coefficients (r) and *p*-values are indicated in each panel.

To verify the clinical relevance of the experimental findings, we extracted NHANES 2007–2010 participants. After applying the exclusion criteria described in methods, a total of 2199 adults were included. They were divided into viral and non-viral hepatitis groups based on the diagnosis of viral hepatitis. Each group was further divided into MASLD and non-MASLD subgroups according to FLI (Figure 1). Baseline characteristics are listed in Supplementary Table S1. As shown in Supplementary Table S1, the viral-hepatitis group was older (median age 55 vs. 48 years) and contained a higher proportion of Mexican-American participants (24.9% vs. 5.0%) than the non-viral group. Within both groups, participants with MASLD (FLI ≥60) had higher BMI, waist circumference, ALT, AST, TG, GGT and FLI values than those without MASLD (FLI <60) (all *P* < 0.001). Spearman correlation showed positive associations between markers of liver injury and lipid variables among participants with both viral hepatitis and MASLD. Serum ALT correlated with FLI (r = 0.329, *P* < 0.001, Figure 9A) and with TG (r = 0.203, *P* < 0.001, Figure 9C). AST also correlated with FLI (r = 0.135, *P* < 0.001, Figure 9B) and with TG (r = 0.100, *P* = 0.002, Figure 9D), although the coefficients were lower than for ALT. These clinical data indicate that greater hepatic lipid accumulation, reflected by higher FLI and TG levels, accompanies increased hepatocellular injury in patients with viral hepatitis and MASLD, supporting the experimental observation that STING-mediated lipotoxicity promotes hepatocyte death.

## 4 Discussion

**FIGURE 10:**
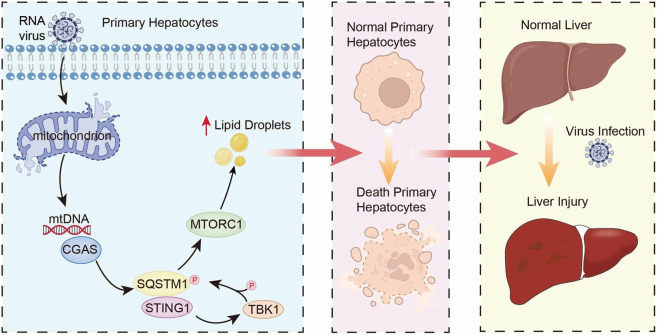
A diagram of the mechanism by which STING exacerbates lipotoxicity in viral infected primary hepatocytes and liver damage related to viral hepatitis.

This study elucidates the mechanistic role of the STING signaling pathway in driving hepatocellular death and liver injury. While STING expression is typically abundant in NPCs and low in quiescent hepatocytes, we observed a distinct, stress-induced upregulation of STING within hepatocytes following VSV infection. Verified in both primary cultures and an *In Vivo* viral hepatitis model, this pathological surge in STING activity progressively intensified over the course of infection. Mechanistically, and corroborating our previous work linking STING to mTOR signaling (Liu et al., 2022), we found that heightened STING activation potentiates mTOR activity. This signaling axis was concomitant with the accumulation of LDs and ferroptosis-associated cellular damage. Crucially, pharmacological blockade of STING using H-151 not only attenuated LD deposition and cytotoxicity *In Vitro* but also ameliorated hepatic pathology, reduced serum transaminase levels, and extended survival in infected mice. Collectively, these findings underscore the maladaptive role of hepatocyte-specific STING activation in viral liver injury and validate STING inhibition as a viable therapeutic strategy (Figure 10).

While the link between lipid metabolism and ferroptosis is well-documented (Pope and Dixon, 2023), the upstream signaling mechanisms driving this interplay in hepatocytes have remained elusive. This study is the first to uncover a central role of the STING signaling pathway in hepatocellular lipid metabolic dysregulation and ferroptosis, providing a novel perspective on the function of STING in hepatocyte death and liver injury. By integrating *In Vitro* viral-infected hepatocyte models with *In Vivo* virus-induced liver injury models, we established a causal relationship between STING activation, LDs accumulation, and ferroptosis, offering new insights into the molecular mechanisms underlying liver failure. Furthermore, our findings validate the therapeutic potential of pharmacological inhibition of STING, such as with the STING inhibitor H-151, in mitigating liver injury, thus laying an experimental foundation for the development of STING-targeted therapies for viral hepatitis and acute liver failure. Currently, significant research efforts are being directed toward targeting the STING pathway as a potential therapeutic strategy for treating various forms of liver injury (Li et al., 2022). The findings of this study may broaden the application scope of STING-targeting therapeutics. This experimental evidence is clinically corroborated by NHANES data: in patients with comorbid viral hepatitis and MASLD, hepatic steatosis and triglyceride levels correlate linearly with injury markers, confirming that metabolic dysfunction synergizes with viral insults to accelerate liver damage. Although this study has made significant progress, there are still some limitations that require further improvement. First, the relationship between the low expression of STING in hepatocytes and the pathological state of liver injury still needs to be further verified. Although there has been an ongoing debate regarding STING expression in hepatocytes, the Human Protein Atlas (HPA) data provide strong evidence that STING1 expression is minimal in human hepatocytes compared to non-parenchymal liver cells and lymphoid tissues. This low physiological baseline supports our hypothesis that the distinct upregulation of STING observed following VSV infection represents a pathological ‘gain-of-function’ event. Our previous study demonstrated that STING is expressed at very low levels in hepatocytes and its expression is upregulated only under specific physiological conditions (Liu et al., 2022). We propose that this low basal expression serves as an essential protective threshold during liver homeostasis. Given that hepatocytes are metabolic hubs frequently exposed to transient oxidative stress and mtDNA fluctuations, maintaining a ‘quiet’ STING profile prevents the unnecessary triggering of the STING-mTOR axis. This ensures that basal lipophagy, a critical process for the lysosomal degradation of triglycerides, remains functional under steady-state conditions, thereby preventing inappropriate lipid accumulation and subsequent lipotoxicity. However, the dramatic upregulation of STING upon viral infection represents a pathological ‘maladaptive switch.’ While this surge in STING is intended to orchestrate a robust innate immune response to clear the virus, its sustained activation in hepatocytes inadvertently hijacks the cellular metabolic machinery. By over-potentiating mTOR activity and impairing lipophagic flux, STING transforms from a protective immune sentinel into a driver of metabolic dysregulation. This “maladaptive switch” results in the accumulation of pro-ferroptotic lipid droplets and lethal lipid peroxidation, ultimately leading to the very hepatocellular death and liver failure it was meant to prevent. It remains to be confirmed whether an upregulation of STING expression is observed in liver tissue from patients with chronic liver injury or cirrhosis, and whether this upregulation is associated with lipid metabolic dysregulation and ferroptosis, which should be confirmed through pathological sample analysis. Second, our previous study found that a high-fat environment induces mitochondrial damage, leading to the release of mitochondrial DNA and subsequent activation of STING. Activated STING enhances mTOR activity via the TBK1-P62 axis, thereby inhibiting the autophagic degradation of LDs (Liu et al., 2022). Now, this study reveals that STING also promotes LDs accumulation through the mTOR signaling pathway in the condition of viral infection. Additionally, the long-term efficacy and safety of the STING inhibitor H-151 also need to be further assessed. Recent studies have shown that the STING inhibitor H-151 can alleviate sepsis-induced acute liver injury (Li et al., 2022), further supporting our findings in this study. Although H-151 significantly improves liver injury in the short term, its potential side effects in long-term treatment and the long-term impact on the immune system need to be validated through chronic liver injury models. Besides, we utilized VSV as a viral stressor to model hepatocyte injury in this study. Previous studies have established that VSV infection primarily triggers the RIG-I-like receptor (RLR) pathway upon sensing viral RNA in the cytosol. However, our findings align with emerging evidence that RNA viruses also induce secondary activation of the cGAS-STING pathway by causing mitochondrial damage and the subsequent release of mtDNA into the cytoplasm (West et al., 2015). Meanwhile, there is still limitation regarding this model. While both viruses cause lipid accumulation, the mechanisms differ: HCV core proteins directly localize to the surface of lipid droplets (LDs) and hijack them as assembly platforms for viral progeny (Miyanari et al., 2007), while also impairing host lipid secretion. In contrast, our findings suggest that in an acute viral stress environment, STING activation impairs lipophagy via the mTOR pathway, leading to pathological LD accumulation and subsequent ferroptotic damage. This study highlights a novel ‘stress-response’ mechanism of lipotoxicity that may synergize with the virus-specific metabolic hijacking seen in chronic hepatitis. Additionally, a limitation of our NHANES analysis is the confounding role of BMI. Since participants with MASLD naturally exhibit higher BMI, it is challenging to entirely isolate the impact of hepatic lipotoxicity from the systemic metabolic effects of obesity on liver enzymes. Although our study demonstrates a clear association between lipid markers and liver injury in viral-infected patients, future prospective studies utilizing multivariate models to adjust for BMI and body composition are warranted to more precisely define the independent contribution of the STING-lipotoxicity axis to viral hepatitis progression. Finally, regarding the epidemiological analysis, the cross-sectional nature of the NHANES dataset precludes causal inferences between lipid accumulation and liver injury progression. Moreover, the lack of direct histological data in NHANES necessitates future longitudinal studies utilizing liver biopsies to definitively link STING upregulation with hepatic steatosis and disease severity. To overcome the limitations of invasive biopsies, future research should also explore non-invasive surrogate markers, such as circulating CXCL10 or cGAMP levels, to assess STING pathway activity in patients. Additionally, advanced imaging techniques like MRI-PDFF or transient elastography could be utilized to more precisely monitor the independent contribution of the STING-lipotoxicity axis to the progression of viral hepatitis.

In summary, by integrating *In Vitro* mechanistic dissection, *In Vivo* disease modeling, and clinical epidemiological analysis, this study elucidates the pivotal role of STING signaling in driving viral-associated hepatocellular injury. These findings substantiate STING inhibition as a viable therapeutic strategy, offering a novel intervention point for metabolic-viral liver pathologies. To translate these preclinical benchmarks into clinical practice, future investigations must prioritize validating the correlation between STING activity and hepatic damage in human biopsy cohorts, as well as delineating the precise molecular crosstalk between STING and lipid metabolism. Furthermore, establishing the chronic safety profile of STING inhibitors remains a prerequisite for developing precise, durable treatments for liver failure.

## Data Availability

The original contributions presented in the study are included in the article/Supplementary Material, further inquiries can be directed to the corresponding authors.
