# Supplementary material for: Inhibition of STING alleviates lipotoxicity in viral-infected primary mouse hepatocytes and viral hepatitis-associated liver damage
Source: Front Pharmacol. 2026 Apr 28;17:1804426. doi: 10.3389/fphar.2026.1804426 (PMC13161481; doi:10.3389/fphar.2026.1804426)
Supplement: Supplementary file 1 [file Table1.docx]

**Supplemental Table S1 Comparison of baseline characteristics in the non-viral hepatitis and viral hepatitis groups**

| characteristic | non-viral hepatitis(N=1214) | | | | viral hepatitis (N=985) | | | |
| --- | --- | --- | --- | --- | --- | --- | --- | --- |
|  | Overall(N=1214) | non-MASLD(N=744) | MASLD(N=470) | *P*-value | Overall(N=985) | non-MASLD(N=604) | MASLD(N=381) | *P*-value |
| Age, years | 48.00 (35.00 - 61.00) | 45.00 (32.00 - 60.00) | 51.00 (39.00 - 61.00) | <0.001 | 55.00 (40.00 - 67.00) | 53.00 (36.00 - 67.00) | 57.00 (44.00 - 66.00) | 0.002 |
| Gender, n (%) |  |  |  | <0.001 |  |  |  | 0.031 |
| Male | 600(49.42) | 325 (43.68) | 275 (58.51) |  | 534 (54.21) | 311 (51.49) | 223 (58.53) |  |
| Female | 614 (50.58) | 419 (56.32) | 195 (41.49) |  | 451 (45.79) | 293 (48.51) | 158 (41.47) |  |
| Race/ethnicity, n (%) |  |  |  | 0.063 |  |  |  | 0.032 |
| Mexican American | 61 (5.02) | 32 (4.30) | 29 (6.17) |  | 245 (24.87) | 132 (21.85) | 113 (29.66) |  |
| Other Hispanic | 57 (4.70) | 40 (5.38) | 17 (3.62) |  | 163 (16.55) | 100 (16.56) | 63 (16.54) |  |
| Non-Hispanic White | 821 (67.63) | 513 (68.95) | 308 (65.53) |  | 338 (34.31) | 217 (35.93) | 121 (31.76) |  |
| Non-Hispanic Black | 240 (19.77) | 134 (18.01) | 106 (22.55) |  | 17.00 (17.87) | 109 (18.05) | 67 (17.59) |  |
| Other Race | 35 (2.88) | 25 (3.36) | 10(2.13) |  | 63 (6.40) | 46 (7.62) | 17 (4.46) |  |
| Education levels, n (%) |  |  |  | 0.019 |  |  |  | <0.001 |
| <High school | 128(10.54) | 71(9.54) | 57(12.13) |  | 278(28.22) | 161(26.66) | 117(30.71) |  |
| High school | 260(21.42) | 151(20.30) | 109(23.19) |  | 201(20.41) | 110(18.21) | 91(23.88) |  |
| Some college or above | 826(68.04) | 522(70.16) | 304(64.68) |  | 506(51.37) | 333(55.13) | 381(45.41) |  |
| BMI, kg/m2 | 27.44 (23.85 - 32.01) | 24.65 (22.16 - 26.94) | 33.20 (30.34 - 37.20) | <0.001 | 27.32 (24.28 - 31.11) | 25.06 (22.67 - 27.16) | 32.15 (29.67 - 35.87) | <0.001 |
| waist circumference, cm | 96.75 (86.10 - 108.00) | 88.70 (81.45 - 95.70) | 110.50 (104.60 - 120.00) | <0.001 | 96.90 (88.40 - 105.70) | 91.00 (82.95 - 96.25) | 108.30 (102.70 - 116.40) | <0.001 |
| ALT, U/L | 21.00 (17.00 - 27.00) | 19.00 (16.00 - 24.00) | 25.00 (19.00 - 34.00) | <0.001 | 21.00 (17.00 - 30.00) | 20.00 (16.00 - 25.00) | 26.00 (20.00 - 36.00) | <0.001 |
| AST, U/L | 23.00 (20.00 - 27.00) | 22.00 (19.00 - 26.50) | 24.00 (20.00 - 29.00) | <0.001 | 24.00 (21.00 - 28.00) | 23.00 (20.00 - 26.00) | 25.00 (21.00 - 30.00) | 0.025 |
| TG, mg/dL | 105.00 (73.00 - 151.00) | 86.00 (63.00 - 118.00) | 143.00 (106.00 - 200.00) | <0.001 | 109.00 (78.00 - 152.00) | 91.00 (67.50 - 121.00) | 148.00 (109.00 - 195.00) | <0.001 |
| GGT, U/L | 20.00 (14.00 - 29.00) | 16.00 (12.00 - 23.00) | 27.00 (19.00 - 39.00) | <0.001 | 21.00 (15.00 - 31.00) | 18.00 (14.00 - 25.00) | 29.00 (20.00 - 43.00) | <0.001 |
| FLI | 44.98 (17.08 - 79.09) | 22.59 (9.40 - 39.66) | 84.72 (73.68 - 94.43) | <0.001 | 47.48 (21.17 - 76.31) | 27.36 (11.96 - 42.35) | 83.52 (72.51 - 91.97) | <0.001 |
